# Astragaloside IV reduces mutant Ataxin-3 levels and supports mitochondrial function in Spinocerebellar Ataxia Type 3

**DOI:** 10.1038/s41598-024-77763-2

**Published:** 2024-10-29

**Authors:** Yongshiou Lin, Wenling Cheng, Juichih Chang, Yuling Wu, Mingli Hsieh, Chinsan Liu

**Affiliations:** 1 Vascular and Genomic Center, Institute of ATPChanghua Christian Hospital, Changhua, Taiwan; 2 Center of Regenerative Medicine and Tissue Repair, Institute of ATPChanghua Christian Hospital, Changhua, Taiwan; 3 General Research Laboratory of Research Department, Changhua Christian Hospital, Changhua, Taiwan; 4 Cardiovascular and Mitochondrial Related Disease Research CenterHualien Tzu Chi HospitalBuddhist Tzu Chi Medical Foundation, Hualien, Taiwan; 5 Department of Life Science, Tunghai University, Taichung, Taiwan; 6 Department of Neurology, Changhua Christian Hospital, 7F., No.235, Syuguang Rd., Changhua, Taiwan; 7 Graduate Institute of Integrated Medicine, China Medical University, Taichung, Taiwan; 8 Department of Post-Baccalaureate MedicineCollege of Medicine, National Chung Hsing University, Taichung, Taiwan

**Keywords:** Spinocerebellar ataxia type 3, Astragaloside IV, Oxidative stress, Autophagy, Mitochondrial dysfunction, Neuroscience, Neurology

## Abstract

This study investigated the therapeutic effects of astragaloside IV (AST) on spinocerebellar ataxia type 3 (SCA3), also known as Machado-Joseph disease (MJD), a neurodegenerative disorder. Human neuroblastoma SK-N-SH cells expressing mutant ataxin-3 protein with 78 CAG repeats (MJD78) were employed as an in vitro model. Protein expression analysis demonstrated that AST treatment reduced mutant ataxin-3 protein expression and aggregation by enhancing the autophagic process in MJD78 cells. Elevated oxidative stress levels in MJD78 cells were significantly reduced following AST treatment, which also enhanced antioxidant capacity, as evidenced by flow cytometry and antioxidant enzyme activity assays. Furthermore, AST treatment ameliorated mitochondrial dysfunction in MJD78 cells, including improvements in mitochondrial membrane potential, respiration, and mitochondrial dynamics. In conclusion, AST administration increased antioxidant capacity, reduced both cellular and mitochondrial oxidative stress, and improved mitochondrial quality control processes through fusion, fission, and autophagy. These mechanisms collectively reduced intracellular mutant ataxin-3 protein aggregation, thereby achieving therapeutic efficacy in the SCA3 model.

**Supplementary Information:**

The online version contains supplementary material available at 10.1038/s41598-024-77763-2.

## Introduction

Spinocerebellar ataxia type 3 (SCA3), also known as Machado-Joseph disease (MJD), is the most common dominantly inherited ataxia, with no effective treatment currently available. Pathologically, SCA3 is caused by an expansion of the cytosine–adenine–guanine (CAG) repeat in the *ATXN3* gene. Healthy populations have up to 40 CAG repeats, whereas individuals who develop SCA3 typically have between 55 and 80 repeats^1^. This mutation results in the production of ataxin-3, a polyglutamine (polyQ) protein prone to aggregation and deposition in cerebellar and brainstem neurons, which ultimately leads to neuronal cell loss^2^. Numerous scholars have identified autophagic dysfunction^3^,^4^ impaired oxidative stress^5,6^, and mitochondrial bioenergetics^7,8^ as the hallmarks of polyQ disease.

Autophagy is a complex pathway that plays an essential role in maintaining cellular homeostasis in neurodegenerative diseases. Through autophagy, aggregated and misfolded proteins are degraded to maintain cellular homeostasis. However, when autophagy is dysfunctional, damaged or dysfunctional components cannot be properly degraded or cleared, resulting in the accumulation of abnormal protein toxicity. Moreover, Moreover, the aggregation of ataxin-3 protein further impairs autophagic function^9^. Beclin1, p62, and Light Chain 3 (LC3), which are key proteins involved in autophagy, have been demonstrated to be dysregulated in SCA3-diseased brains in vivo and in vitro models^10,11^.

Numerous research reports have suggested that oxidative stress plays a key role in the pathogenesis of neurodegenerative diseases^12,13^. Oxidative stress arises from an imbalance between the production and clearance of reactive oxygen species (ROS), leading to potential cellular damage. Protein misfolding and aggregation can exacerbate ROS production^14^, resulting in damage to cellular components. In addition, the generation of ROS is linked to the inactivation of antioxidant systems. Antioxidants, which may be endogenous (produced within the body) or exogenous (acquired from the diet), play crucial roles in protecting against oxidative stress. Key endogenous antioxidants include glutathione (GSH), superoxide dismutase (SOD), and catalase (CAT)^15^. Antioxidants have been demonstrated to reduce aggregation and cell death in various polyQ disease models^16^.

Mitochondrial dysfunction is also considered a pathogenesis of neurodegenerative diseases. Mitochondria, present in all eukaryotic cells, are essential for ROS metabolism, adenosine triphosphate (ATP) production, and apoptosis regulation^17^. Although excessive amounts of ROS can harm mitochondrial energy production, mitochondria are a major source of endogenous ROS during cellular respiration and are therefore essential to various cellular functions^18^. When they are present in moderate amounts, mitochondrial ROS trigger the antioxidant compensation mechanism, thereby contributing to metabolic balance^19^. Several studies have indicated that the brain is an organ that consumes considerable amounts of oxygen and energy and that the oxygen consumption of the brain is impaired in patients with neurodegenerative diseases^20,21^. Furthermore, mitochondrial dynamics are essential for regulating the morphology and primary functions of organelles, as well as their transport through fusion and fission processes^22^. An increase in oxidative stress causes an imbalance of mitochondrial fission and fusion, which is frequently associated with mitochondrial fragmentation, resulting in mitochondrial dysfunction^23^.

Astragaloside IV (AST), a major bioactive compound derived from Astragali Radix (*Huangqi* in Chinese)^24^, has neuroprotective, anti-inflammatory, and neuroregenerative properties^25–27^. The efficacy of AST has also been described in experimental models of various neurodegenerative diseases^28^. AST triggers the induction of autophagy and therefore has an antiapoptotic effect and protects against cartilage degeneration^29^, maintains mitochondrial membrane potential (MMP), increases ATP production^30^, and activates Nuclear factor erythroid 2-related factor 2 (Nrf2) signaling to protect neurons^31^.

To the best of our knowledge, no study has yet evaluated treatment with AST for SCA3. Using a SCA3 cell model, we determined that AST can be used as a medication for treating SCA3 because it reduces mutant ataxin-3 protein levels (including aggregation), reduces oxidative stress, and increases mitochondrial function.

## Materials and methods

### Cell culture and treatment

Human neuroblastoma cell lines (SK-N-SH) stably transfected with the full-length *ATXN3* gene with 26 (MJD26) or 78 CAG repeats (MJD78) were provided by Prof. Mingli Hsieh (Department of Life Science, Tunghai University, Taiwan). The cells were grown in Dulbecco’s modified Eagle medium (DMEM; high glucose, DMEM-HG, GIBCO/Invitrogen, Carlsbad, CA, USA) containing 10% heat-inactivated fetal bovine serum (HyClone Laboratories, Logan, UT, USA), 1% penicillin/streptomycin (GIBCO, Thermo Fisher Scientific, Inc., Waltham, MA, USA), 1% L-glutamine (GIBCO), 1% non-essential amino acids (GIBCO), and 100-µg/mL G418 (InvivoGen, San Diego, CA, USA) at 37 °C in a humidified atmosphere of 5% CO_2_. The culture medium was replaced every 2–3 days. The cells were allowed to adhere and grow to 85% confluency for 24 h before experiments were performed. AST (purity: high-performance liquid chromatography value > 98%, U-04410-8-100MG; Sigma-Aldrich, St. Louis, MO, USA) was dissolved in dimethylsulfoxide (DMSO) to the concentration of 80 mg/mL as a stock solution. The maximum final concentration of DMSO in the medium was maintained at 0.1% to ensure the cell viability would not be affected. The cells were exposed to various doses (0, 12.5, 25, 50 µM) of AST for 24 h.

### Protein aggregation assay

Aggregation was measured using PROTEOSTAT Protein Aggregation Assay kit (ENZ-51023-KP002, Enzo Life Sciences, PA, USA) per the manufacturer’s instructions^32,33^. In brief, after being treated for 24 h, the cells were washed with phosphate-buffered saline (PBS), trypsinized with 0.05% trypsin, and collected into PBS. Diluted PROTEOSTAT detection reagent was dispensed into 96-well microplates, after which the protein of interest was added to each well and incubation was performed in the dark for 15 min at room temperature (RT). Fluorescence intensity was measured using a CLARIOstar Plus microplate reader (https://reurl.cc/WxWoZ5; BMG LABTECH, Offenburg, Germany); the reader’s excitation wavelength and emission filter were set to approximately 550 and 600 nm, respectively.

### Western blot analysis

The cells were treated for 24 h, washed with PBS, and trypsinized with 0.05% trypsin, after which their cell lysates were extracted using Pierce IP Lysis Buffer (#87788; Thermo Scientific, MA, USA) supplemented with the protease inhibitor cocktail Set I (#539131; Calbiochem, Billerica, MA) and Phosphatase Inhibitor Cocktail Set V 50x (#524629, Merck Millipore, MA USA). After the cells were lysed on ice for 30 min, they were centrifuged at 14,000 rpm and 4 °C for 30 min. The supernatants were collected, and the protein concentration was determined using the Pierce BCA Protein Assay Kit (#23227, Thermo Scientific). The obtained samples were mixed with AllBio 6X SDS Loading Dye (AllBio Science, ABMBD-001, Taiwan) and denatured at 95 °C for 5 min. The proteins (20 to 40 µg/lane) were separated using SDS-PAGE gels (10%, #1610183; 12%, #1610185; Bio-Rad, CA, USA), transferred to PVDF membranes (#1704272; Bio-Rad), blocked with blockEBL blocking buffer (MPR-02100, Taipei, Taiwan) for 1 h at RT, and incubated with the following primary antibodies overnight at 4 °C: optic atrophy 1 (OPA1, #612607, 1:500; BD Biosciences, San Jose, CA, USA), mitofusin 2 (MFN2, M6319, 1:500; Sigma-Aldrich), p-Drp1 (#3455, 1:250; Cell Signaling, Danvers, MA, USA), dynamin-related protein 1 (Drp1, ab154879, 1:500; Abcam, Cambridge, MA, USA), mitochondrial fission protein 1 (Fis1, ab71498, 1:500; Abcam), translocase of inner mitochondrial membrane 23 (Tim23, #611222, 1:500; BD Biosciences), glyceraldehyde 3-phosphate dehydrogenase (GAPDH, ab9484, 1:1000; Abcam), ataxin-3 (ab175265, 1:500; Abcam), Beclin1 (NB110-87318, 1:1000; Novus Biologicals, Littleton, CO, USA), p62 (ab56416, 1:1000; Abcam), and LC3 (Cell Signaling, #4108, 1:500). The obtained membranes were washed with tris-buffered saline with Tween 20 and incubated for 1 h with HRP-conjugated secondary antibodies at RT (1:10000; Jackson Immuno Research Laboratories, PA, USA). After the membranes were washed, signals were visualized using the Immobilon Western Chemiluminescence HRP substrate (WBKLS0500, Merck Millipore) and a Fusion-FX7-826.WL Superbright Transilluminator (Vilber Lourmat, Eberhardzell, Germany). The obtained bands were quantified using the imaging processing software ImageJ (Version 1.48; https://imagej.net/ij/; Rasband, W.S., National Institutes of Health, Bethesda, MD, USA).

### Cell viability

MJD26 (15000/well) and MJD78 (20000/well) cells were seeded in transparent 96-well plates and incubated overnight. The cells were then treated with various concentrations of AST (12.5, 25, 50, or 100 µM) and incubated for 24 h. After the medium was removed, the cells were incubated in fresh medium with 10% WST-1 reagent (11644807001, Roche, Basel, Switzerland) for 3 h. Absorbance was measured at 450 nm by using a multiplate reader, and a reference wavelength of 690 nm was used; the absorbance value at 690 nm was subtracted from that at 450 nm. The relative cell viability percentage in each group was calculated using comparisons with the cell viability of the control group.

### Cell apoptosis

The percentage of apoptosis was estimated using FlowCellect^®^ Kit (FCCH100106, Millipore). MJD26 and MJD78 cells were seeded in 12-well plates with a density of 4 × 105 cells/well. After 24 h of treatment, the cells were suspended in 1× assay buffer, staining with Annexin V (detection of early apoptotic cells) and 7-amino actinomycin D (a membrane-impermeant dead cell dye) and detected cells were measured using the NucleoCounter^®^ NC-3000™ fluorescence advanced image cytometer (https://chemometec.com/nucleocounters/nc-3000/; ChemoMetec, Denmark) and analyzed immediately by NucleoView™ software (https://chemometec.com/software/nucleoview/; ChemoMetec, Denmark).

### Oxidative stress and mitochondrial membrane potential

The cells were treated with AST per the procedure as described above. Dihydrofluorescein diacetate (DCF, 1 µM; Invitrogen, Waltham, MA, USA) staining, dihydroethidium (DHE, 10 µM; Invitrogen) staining, MitoSOX Red (5 µM; Invitrogen) staining, and tetramethylrhodamine ethyl ester perchlorate (TMRE, 100 nM; Invitrogen) staining were used to quantify total ROS, mitochondrial superoxide, superoxide ROS, and MMP, respectively. Fluorescence intensity was measured using a BD FACSCalibur flow cytometer (FC500, Beckman Coulter, Miami, FL, USA).

### Antioxidant enzyme activity assay

The cells were treated with AST per the procedure described in a previous subsection. Total GSH, SOD, glutathione peroxidase (GPx), and CAT were measured using a total GSH colorimetric assay kit (E-BC-K097-M; Elabscience Biotechnology, TX, USA), an Amplite colorimetric SOD assay kit (#11305; AAT Bioquest, Sunnyvale, CA, USA), a GPx activity assay kit (E-BC-K096-M; Elabscience Biotechnology), and a CAT activity assay kit (E-BC-K031-M; Elabscience Biotechnology), respectively, per the manufacturers’ instructions; detection was performed using a CLARIOstar microplate reader (https://reurl.cc/WxWoZ5; BMG LABTECH, Offenburg, Germany).

### Mitochondrial respiration

The respiration rates of mitochondria in MJD26 and MJD78 cells were measured at 37 °C using a high-resolution Oroboros Oxygraph 2 K respirometer (O2k; Oroboros, Innsbruck, Austria) per the procedure described in another study^34^. In brief, 0.5-mM malate and 10-mM L-glutamate were added to obtain the basal respiration rate of the mitochondrial electron transport chain (Routine), 2.5-mM adenosine diphosphate was added to induce complex I–linked oxidative phosphorylation (OXPHOS), 10-mM succinate was added to enable observation of CI + II-linked OXPHOS (maximum OXPHOS [Max-Ox]), 5-µM oligomycin was added to inhibit ATP synthase, and 1.5-µM FCCP was added to determine the maximum noncoupled respiration (Max-U). Finally, 0.5-µM rotenone and 5-µM antimycin A were added to block respiratory electron flux at mitochondrial complexes I and III to completely shut down mitochondrial oxygen consumption and enable measurement of residual oxygen consumption (ROX).

### Statistical analysis

All experiments were conducted at least in three independently technical replicates at different times. In vitro data are presented as means ± standard deviations (SD). Graphics were generated using the GraphPad Prism software (version 7.0; https://www.graphpad.com/features, GraphPad Prism Software, San Diego, CA, USA). Statistical analysis was conducted using a one-way analysis of variance (ANOVA), followed by LSD as a post hoc test. All statistical comparisons were made using SPSS 17.0 software (SPSS, Chicago, IL, USA). A value of *p* < 0.05 was regarded as statistically significant, and such values are indicated in the figures with an asterisk.

## Results

### Effects of AST on protein aggregation and autophagic clearance of mutant ataxia-3

**Fig. 1:**
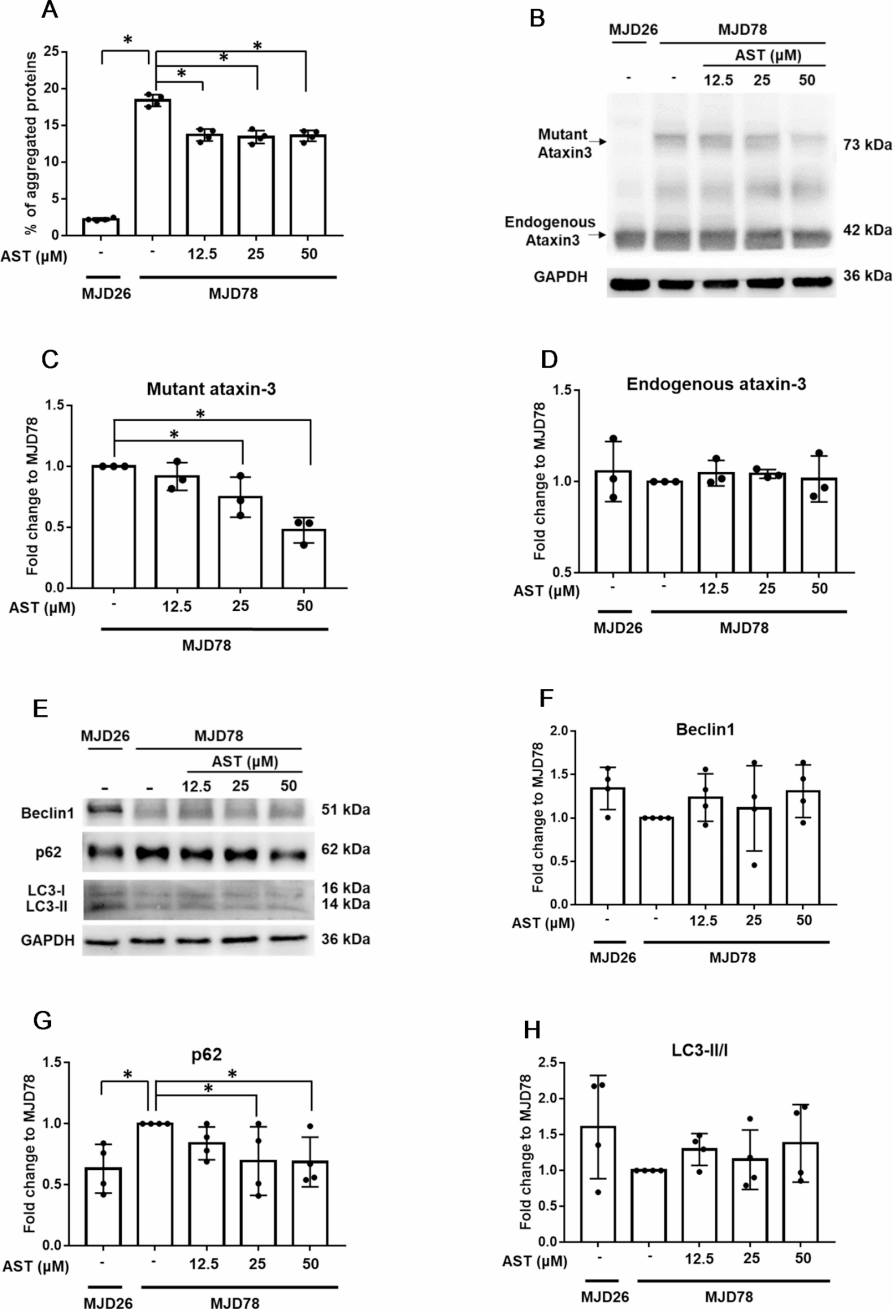
The levels of protein aggregation, ataxin-3 protein (both mutant and endogenous), and autophagy-related proteins in Machado-Joseph Disease cells after treatment with different concentrations of astragaloside IV for 24 h. (**A**) Protein aggregation was assessed using a protein aggregation detection kit (*N* = 4). (**B-D**) The expression levels of mutant and endogenous ataxin-3 proteins (*N* = 3), as well as (**E-H**) autophagy markers [Beclin1 (*N* = 4), p62 (*N* = 4), and LC3-II/I (*N* = 4)], were analyzed through Western blotting. Protein quantification was normalized to GAPDH expression levels. The average of independent replicates was shown as points bar, with error bars indicating the standard deviation. *p < 0.05 was considered significant. Abbreviation: astragaloside IV, AST; Machado-Joseph Disease, MJD; SK-N-SH with 26 CAG repeats, MJD26; SK-N-SH with 78 CAG repeats, MJD78.

To explore the potential of AST for treating SCA3, protein aggregation was assessed using a PROTEOSTAT Protein Aggregation Assay. The results reveal that the MJD78 cells exhibited a higher protein aggregation level than the MJD26 cells. AST treatment significantly reduced the percentage of protein aggregation at all examined dose levels (Fig. 1A). To further confirm the ability of AST to eliminate the mutant form of ataxin-3 protein, protein expression was tested through Western blotting. MJD78 cells expressed the mutant form of ataxin-3 protein, attributed to the expansion of the CAG repeat encoding polyQ. This phenomenon, however, was not observed in MJD26 cells. MJD78 cells expressed the mutant form of ataxin-3 protein, attributed to the expansion of the CAG repeat encoding polyQ. This phenomenon, however, was not observed in MJD26 cells. The incubation of MJD78 cells with AST resulted in a dose-dependent reduction in mutant ataxin-3 protein levels, especially when the dosages were high (Fig. 1B, C), and no significant in endogenous ataxin-3 between groups (Fig. 1D). Compared with the MJD26 cells, the MJD78 cells exhibited reduced levels of autophagy-related protein Beclin1 and reduced LC3-II conversion (LC3-II to LC3-I ratio) and increased p62 accumulation (Fig. 1E). By contrast, the doses of 25 and 50 µM AST significantly decreased the accumulation of p62 (Fig. 1G), and the higher Beclin1 and LC3-II/I ratios were also shown in the treated group but did not reach significant differences (Fig. 1F-H). This finding demonstrates that the increased autophagy induced by 50 µM of AST was related to this dose of AST, leading to more effective removal of mutant protein accumulation.

### Effects of AST on cell viability and oxidative stress

**Fig. 2:**
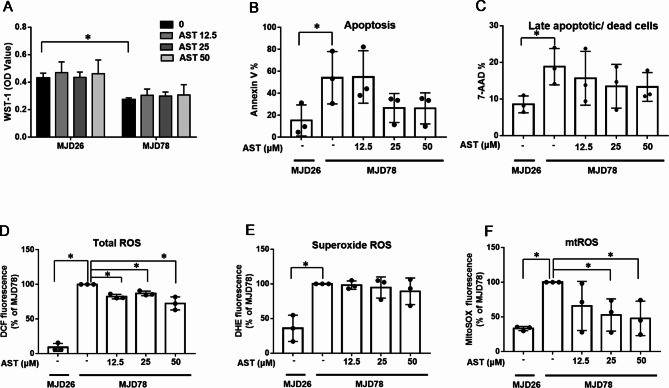
The levels of cell viability and oxidative stress in Machado-Joseph Disease cells after treatment with different concentrations of astragaloside IV for 24 h. (**A**) Cell viability was assessed using the WST-1 assay (*N* = 3), (**B-C**) Apoptotic (*N* = 3) and late apoptotic/ dead cells (*N* = 3)a were stained with annexin V and F by NucleoCounter^®^ NC-3000™ fluorescence image cytometer. Oxidative stress was detected through flow cytometry analysis involving (**D**) DCF staining for intracellular (total) ROS (*N* = 3), (**E**) DHE staining for superoxide ROS (*N* = 3), and (**F**) MitoSOX Red staining for mitochondrial superoxide (mtROS) (*N* = 3). The average of independent replicates was shown as points bar, with error bars indicating the standard deviation. * *p* < 0.05 was considered significant. Abbreviation: astragaloside IV, AST; Machado-Joseph Disease, MJD; SK-N-SH with 26 CAG repeats, MJD26; SK-N-SH with 78 CAG repeats, MJD78.

To assess the cytotoxicity of AST, cells were treated with varying concentrations of AST (0, 12.5, 25, and 50 µM) for 24 h. As shown in the WST-1 assay results (Fig. 2A), MJD78 cells displayed significantly lower viability and no significant viability differences were observed between the MJD26 and MJD78 cells following 24 h of treatment. Cell death analysis using Annexin V/7AAD assays revealed a 3-fold increase in early apoptosis rates (Fig. 2B) and a 2-fold increase in late apoptosis/death rates in MJD78 cells compared to controls with significant differences (Fig. 2C). AST treatment led to slight reductions in both early apoptosis and cell death, although these changes were not statistically significant across groups (Fig. 2B-C). Additionally, MJD78 cells demonstrated a marked increase in oxidative stress relative to MJD26 cells. Following AST treatment, total and mitochondrial ROS levels in MJD78 cells decreased, particularly at the 50 µM concentration (Fig. 2D-F).

### Effects of AST on antioxidant capacity

**Fig. 3:**
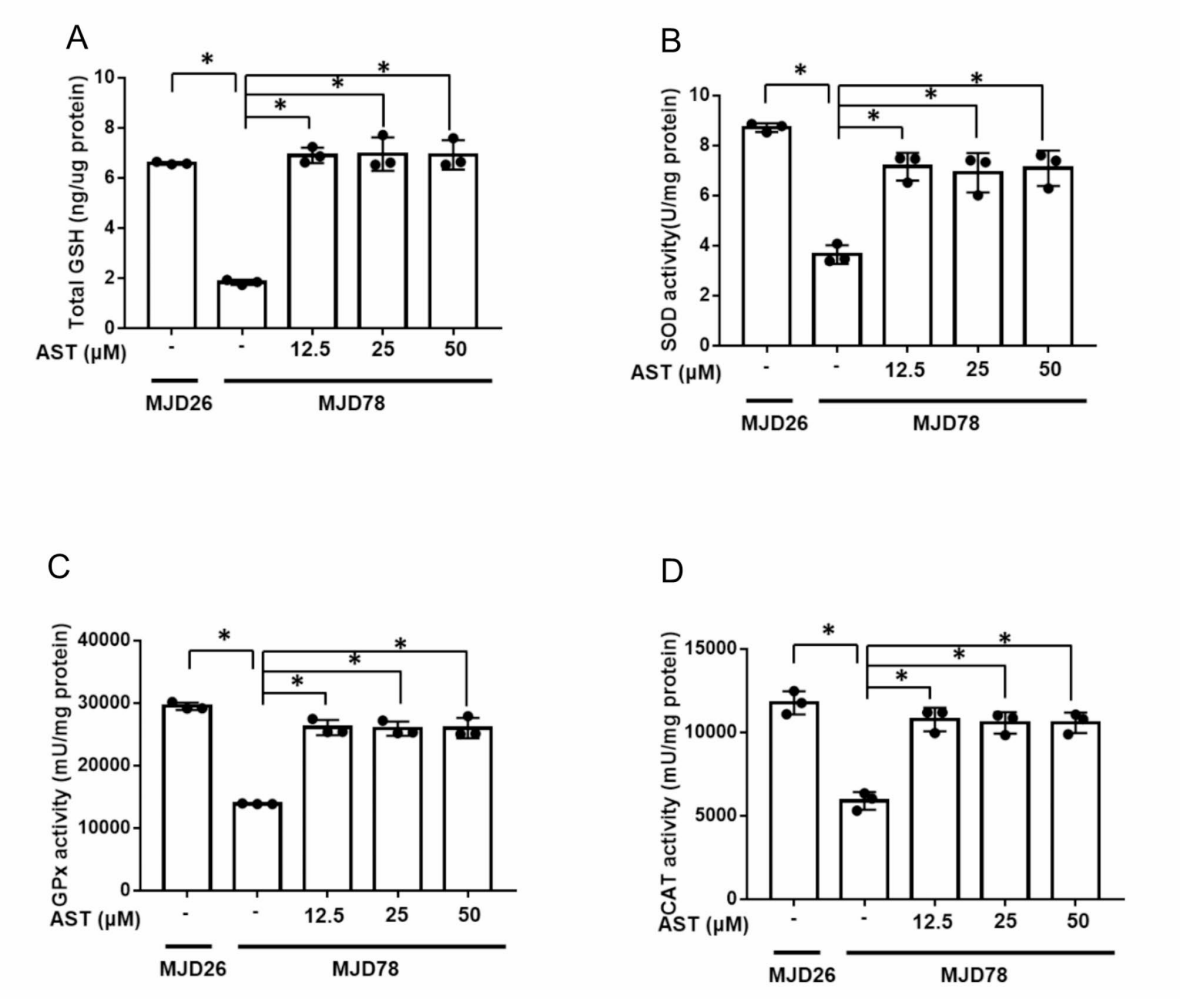
The antioxidant enzymatic activity in Machado-Joseph Disease cells after treatment with different concentrations of astragaloside IV for 24 h. The levels of enzymatic activity, including (**A**) glutathione (GSH) (*N* = 3), (**B**) superoxide dismutase (SOD) (*N* = 3), (**C**) glutathione peroxidase (GPx) (*N* = 3), and (**D**) catalase (CAT) (*N* = 3), were measured using their respective activity assay kits based on the colorimetric method. The average of independent replicates was shown as points bar, with error bars indicating the standard deviation. *p < 0.05 was considered significant. Abbreviation: astragaloside IV, AST; Machado-Joseph Disease, MJD; SK-N-SH with 26 CAG repeats, MJD26; SK-N-SH with 78 CAG repeats, MJD78.

To enable assessment of antioxidant capacity, the MJD78 cells treated with various doses of AST were comprehensively evaluated for enzymatic antioxidants, including total GSH (Fig. 3A), SOD (Fig. 3B), GPx (Fig. 3C), and CAT (Fig. 3D). The data presented in Fig. 3 reveal a considerable decrease in antioxidant system activity in the MJD78 cells. The levels of all antioxidant-related molecules increased consistently and significantly at all tested doses of AST in a non-dose-dependent manner.

### Effects of AST on mitochondrial respiration and dynamic-related proteins

**Fig. 4:**
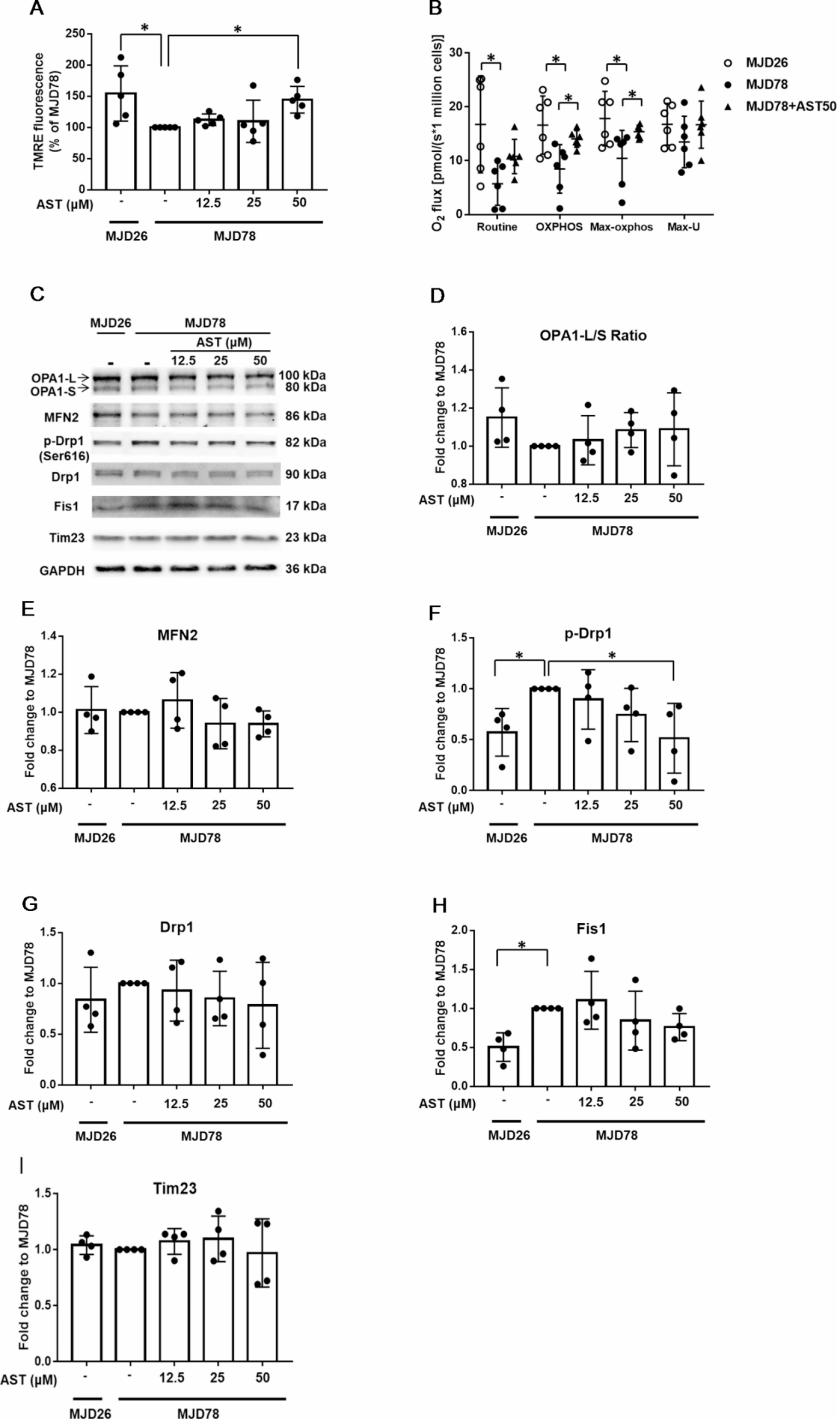
Influence of astragaloside IV on mitochondrial bioenergetics and levels of dynamic-related proteins in Machado-Joseph Disease cells after treatment with various concentrations for 24 h. (**A**) The membrane potential of mitochondria was analyzed by Tetramethylrhodamine ethyl ester intensity using a flow cytometer (*N* = 5). (**B**) Oxygen consumption of cells at different mitochondrial stages was detected by Oroboros 2 K respirometer and the respiratory capa cities were reflected in the routine, oxidative phosphorylation, maximal mitochondrial phosphorylation and maximal mitochondrial uncoupled respiration (*N* = 5). (**C**) The expression level of mitochondrial-related protein. (**D-E**) The expression level of mitochondrial fusion (OPA1 and MFN2) and (**F-H**) fission proteins (Drp1, phosphorylated Drp-1and Fis1), and (**I**) mitochondrial marker protein (Tim23) detected through Western blotting were normalized to the GAPDH protein (*N* = 4). Data was presented as expression levels relative to those of nontreated MJD78 cells. The average of independent replicates was shown as points bar, with error bars indicating the standard deviation. *p < 0.05 was considered significant. Abbreviation: astragaloside IV, AST; Machado-Joseph Disease, MJD; SK-N-SH with 26 CAG repeats, MJD26; SK-N-SH with 78 CAG repeats, MJD78; oxidative phosphorylation, Tetramethylrhodamine ethyl ester, TMRE; OXPHOS; maximal mitochondrial phosphorylation respiration, Max-Ox; maximal mitochondrial uncoupled respiration, Max-U; phosphorylated Drp-1(p-Drp1).

To explore the potential effects of AST on the mitochondrial function of MJD78 cells, we measured the MMP, mitochondrial respiration, and levels of mitochondrial-shaping protein. First, the relative fluorescence of TMRE was used to determine the MMP, and the results reveal a significantly higher TMRE fluorescence in the MJD26 cells than in the MJD78 cells. However, after the cells underwent AST treatment for 24 h, the MMP was revealed to be upregulated in the MJD78 cells, with notable increases observed at AST doses of 50 µM (Fig. 4A). Second, we used O_2_k to measure oxygen consumption (O_2_ flux) and assess the overall mitochondrial respiration in the MJD26 and MJD78 cells. Several quantification parameters of O_2_ flux, including basal respiration (Routine), OXPHOS, and Max-Ox, were significantly lower in the MJD78 cells than in the MJD26 cells; however, this phenomenon was not identified for Max-U. After AST treatment was administered, mitochondrial respiration was notably increased, particularly in OXPHOS and Max-Ox of O2 flux, showing a significant difference (Fig. 4B). Third, we assessed mitochondrial proteins through Western blotting. Mitochondrial dynamics (fusion and fission) maintain the integrity of the mitochondrial network (Fig. 4C). MFN2 and OPA1 are mitochondrial fusion proteins (Fig. 4D-E), whereas Drp1 and Fis1 are mitochondrial fission proteins. The levels of phosphor-Drp1 (S616) and Fis1 proteins were significantly higher in the MJD78 cells than in the MJD26 cells (Fig. 4F-H), whereas the expression of MFN2, OPA1, and Tim23 proteins (Fig. 4I) remained unchanged. After AST treatment was administered, the expression of mitochondrial fission proteins was downregulated, fusion proteins remained largely unchanged (e.g., MFN2), or the ratio increases (e.g., OPA1-L/S) without reaching significance, which may suggest a shift towards pro-fusion to balance mitochondrial dynamic. (Fig. 4C-I).

## Discussion

The therapeutic effects of AST on SCA3 were validated using multiple models. The results consistently indicated that AST treatment provided several benefits, namely reducing protein aggregation and mutant ataxin-3 protein expression, ameliorating mitochondrial function and increasing the antioxidant capacity of MJD78 cells. These mechanisms could be related to the activation of the autophagy-mediated clearance of mutant proteins in the MJD78 cell line during Nrf2 agonist treatment^34^.

PolyQ expansion enhances the aggregation tendency of mutant proteins, with progressive formation of insoluble aggregates being a hallmark of many neurodegenerative diseases^35^. Studies have reported impaired autophagy in the brains of patients with SCA3 and significantly decreased levels of Beclin1 during the initial formation of autophagosomes^10^. In addition, research indicates other autophagy biomarkers (e.g., p62 and LC3) are activated upon stress induction, enabling selective autophagic flux, and LC3 protein plays a crucial role in autophagy in that it facilitates autophagosome elongation and closure^36^. The accumulation of p62 protein is typically regarded as an indicator of autophagy inhibition^37^, and the clearance of mutant ataxin-3 protein through autophagy may be hindered by various factors. In the present study, the protein expression of Beclin1 and the LC3-II/I ratio was lower, and the level of p62 was higher in the MJD78 cells than in the MJD26 cells. After AST treatment was administered, the trends for protein aggregation, mutant ataxin-3 protein expression, and autophagic dysfunction were reversed, suggesting that the beneficial effects of AST could be related to the induction of autophagy in the MJD78 cells. Similarly, Liu et al. reported that AST-mediated autophagy plays an antiapoptotic role, protecting cells from interleukin‑1β‑induced cartilage degeneration^29^. Furthermore, the AST-induced rescue of a lower LC3 signal could be indicative of an increased number of autophagosomes. In our preliminary assessment, we examined their effects on autophagic flux by studying the progression from autophagosome to autolysosome (Supplementary Fig. 1). The results consistently showed that autophagy activity was lower in MJD78 cells than in MJD26 cells, with AST (50 µM) being the most effective in stimulating autophagic flux. Nonetheless, additional experiments are needed to accurately assess the impact of AST treatment on controlling the flow of autophagy, which may involve the use of autophagy inhibitors like chloroquine or BafA1.

ROS production at an appropriate level plays a physiological role in cellular differentiation and proliferation, whereas ROS expression at a level that exceeds physiological limits results in oxidative stress^38^. The overproduction of ROS and impaired antioxidant activity are accelerated by neurodegenerative diseases, which leads to disease progression. Our results indicate that both intracellular and superoxide ROS were significantly increased in the MJD78 cells, this increase was significantly reversed by AST treatment administered at the concentration of 50 µM. Gui et al. demonstrated that AST reduced the high glucose–induced ROS production and oxidative stress in podocytes^39^. In the present study, a high dose of AST (100 µM) increased cellular ROS levels (Supplementary Fig. 2). This finding is consistent with that reported by Sun et al., who employed a neural cell model of Alzheimer’s disease and reported that Treatment concentration of AST at less than 50 µM improved cell survival and reduced Amyloid-β-induced neurotoxicity but had the opposite effect when the concentration was increased to 100 µM^40^. Therefore, when AST is used to treat neurodegenerative diseases, the proper dosage for minimizing potential counter-effects must be considered. Protection against oxidative stress is dependent on endogenous antioxidants, including GSH, SOD, GPx, and CAT. However, neurons have been demonstrated to exhibit a weaker antioxidant defense and limited regenerative capacity relative to other tissue cells^41^. After AST treatment was administered, the levels of antioxidant-related molecules increased, demonstrating that AST can restore antioxidant capacity and potentially counteract the detrimental effects of oxidative stress in individuals with neurodegenerative conditions.

The brain consists of billions of neurons that form complex networks through electrical and chemical signals, making this process highly energy-intensive^42^. Thus, in numerous neurodegenerative disorders, mitochondrial dysfunction has been implicated in SCA3 disease progression^43^. Dysfunctional mitochondria disrupt the key energy production processes within neurons, leading to impaired neuronal function. The key indicators for maintaining mitochondrial quality include the MMP, oxygen consumption rate, and mitochondrial dynamics. The MMP plays a crucial role in the functioning of mitochondria, and it drives ATP synthesis by facilitating the flow of protons^44^. Compared with the fibroblasts from healthy individuals, those from individuals with SCA3 contain fragmented and circular mitochondria in addition to lower levels of OXPHOS complexes, ATP production, and cell viability^7^. This phenomenon is consistent with our results, which indicate that the MMP and level of mitochondrial respiration, including Routine, OXPHOS, and Max-Ox, were significantly lower in the MJD78 cells. Notably, after AST treatment was administered, the MMP and mitochondrial respiration were restored in the cells. In addition, although the effects of AST on mitochondrial dynamics remain unclear, in the present study, the levels of mitochondrial fission proteins, p-Drp1, and Fis1 were significantly higher in the examined MJD78 cells than in the examined MJD26 cells but lower after AST treatment, suggest reduced mitochondrial fission. Furthermore, this AST treatment process did not affect the amount of mitochondria, as indicated by the results regarding the expression of the inner membrane protein Tim23 and mitochondrial DNA copy number (Supplementary Fig. 3). Abnormal mitochondrial dynamics characterized by excessive numbers of mitochondrial fragments has been reported in various models of SCA3^45^,^46^. The processes of mitochondrial fission and fusion have been widely reported to play an essential role in preserving the functionality of mitochondria^47^. A study reported that decreased mitochondrial fragmentation and increased mitochondrial elongation were observed in neural neuroblastoma cells of SCA3 that were treated with far-infrared radiation^45^. The present results reveal that the regulatory effect of AST is related to the regulation of mitochondrial function, including respiratory efficiency and dynamic performance.

**Fig. 5:**
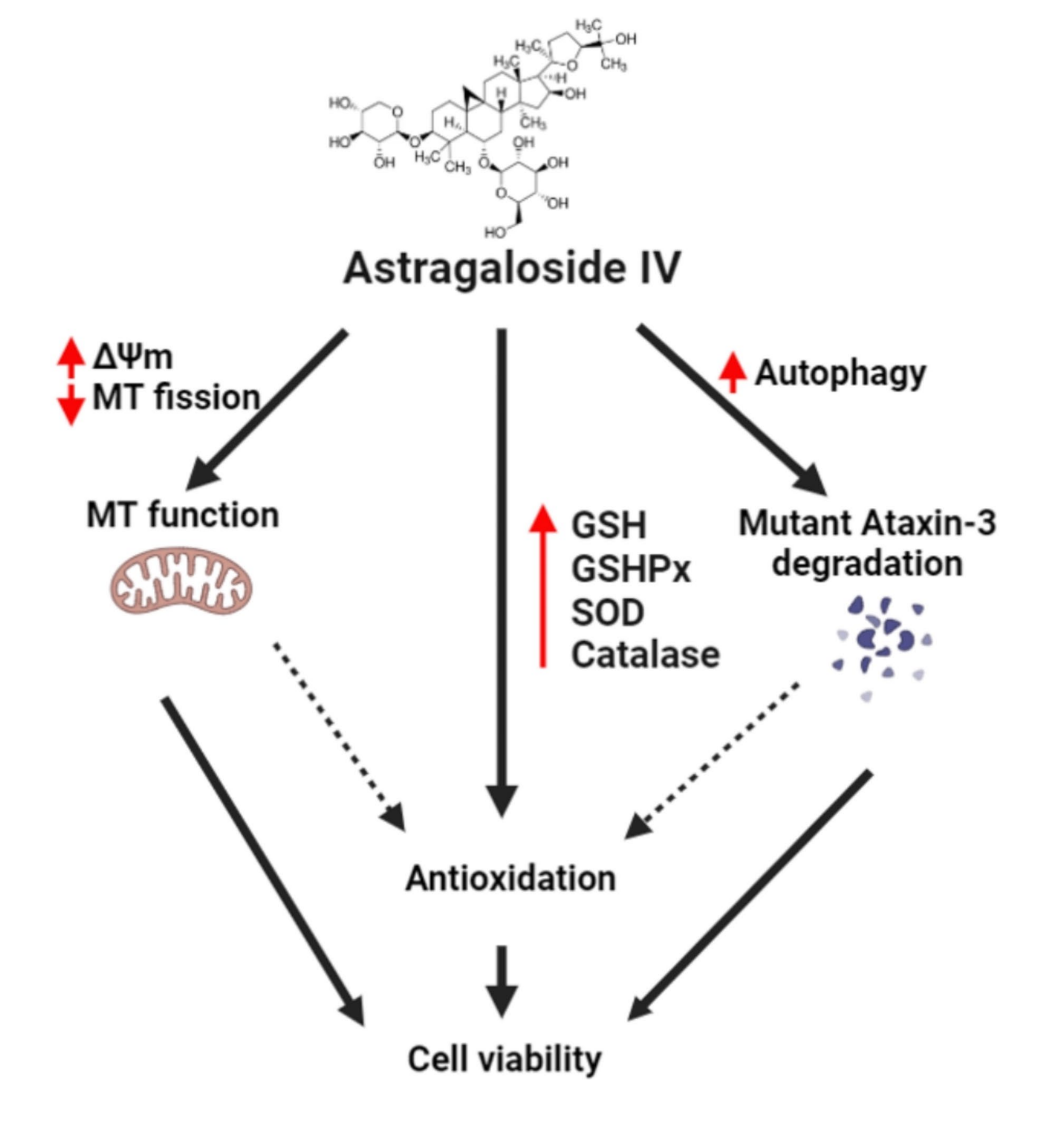
An illustration of astragaloside IV-associated regulation in SCA3. Astragaloside IV treatment supports cell viability by protecting against apoptosis, with therapeutic effectiveness in the SCA3 linked to reduced intracellular mutant ataxin-3 protein aggregation. Moreover, astragaloside IV treatment improves mitochondrial function by reducing mitochondrial ROS, increasing mitochondrial energy metabolism, and decreasing excessive mitochondrial fission. This may be due to its ability to enhance antioxidant enzyme activity in diseased cells, thereby boosting antioxidant capacity. Improvements in mitochondrial function and the reduction of mutant protein aggregation collectively alleviate cellular oxidative stress, contributing to the therapeutic repair of SCA3. Dashed line: interrelated relationship; Solid line: direct regulation.

In summary, the present study provides the effects of AST treatment on SCA3. The mechanism of action of AST is associated not only with decreased protein aggregation and mutant ataxin-3 protein expression but also with decreased oxidative stress, enhanced mitochondrial function, decreasing excessive mitochondrial fission and enhance antioxidant enzyme activity to supports cell viability (Fig. 5). Thus, on the basis of our findings, we suggest the use of AST as a potential therapeutic intervention for impeding SCA3 progression.

## Electronic Supplementary Material

Below is the link to the electronic supplementary material.

Supplementary Material 1

Supplementary Material 2

## Data Availability

The data presented in this study are available on request from the corresponding author.

## Abbreviations

AST: astragaloside IV
ATP: adenosine triphosphate
CAG: cytosine–adenine–guanine
CAT: catalase
DCF: Dihydrofluorescein diacetate
DHE: dihydroethidium
Drp1: dynamin-related protein 1
Fis1: fission protein 1
GAPDH: glyceraldehyde 3-phosphate dehydrogenase
GPx: glutathione peroxidase
GSH: glutathione
H&E: hematoxylin and eosin
LC3: Light Chain 3
Max-U: maximum noncoupled respiration
MFN2: mitofusin 2
MJD: Machado-Joseph disease
MMP: mitochondrial membrane potential
Nrf2: Nuclear factor erythroid 2-related factor 2
OPA1: optic atrophy 1
OXPHOS: oxidative phosphorylation
PBS: phosphate-buffered saline
PCR: Polymerase chain reaction
polyQ: polyglutamine
ROS: reactive oxygen species
ROX: residual oxygen consumption
RT: room temperature
SCA3: spinocerebellar ataxia type 3
SK-N-SH: Human neuroblastoma cell lines
SOD: superoxide dismutase
Tim23: inner mitochondrial membrane 23
TMRE: tetramethylrhodamine ethyl ester perchlorate
