# Supplementary material for: Astragaloside IV reduces mutant Ataxin-3 levels and supports mitochondrial function in Spinocerebellar Ataxia Type 3
Source: Sci Rep. 2024 Oct 29;14:25979. doi: 10.1038/s41598-024-77763-2 (PMC11522510; doi:10.1038/s41598-024-77763-2)
Supplement: Supplementary file 1 — Supplementary Material 1 [file 41598_2024_77763_MOESM1_ESM.docx]

Figure 1. Expression of protein aggregation, ataxin-3 protein, and autophagy-related proteins. Cells were treated with different concentrations of AST for 24 h.

C

B


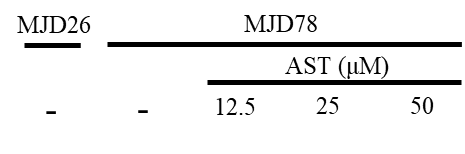

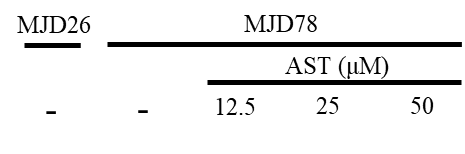


GAPDH


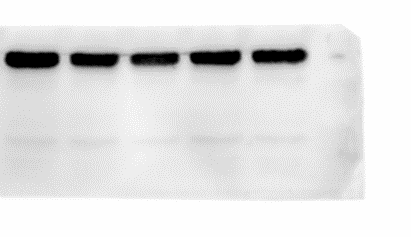

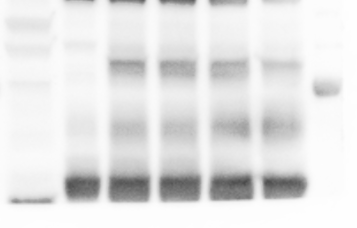

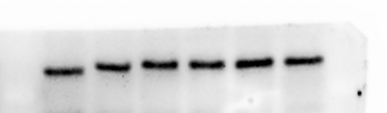

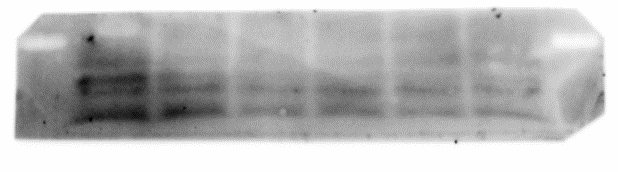

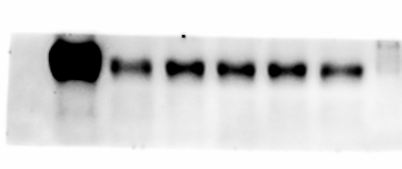

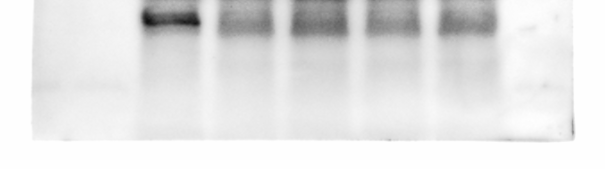


GAPDH

LC3I

LC3II

Beclin1

P62

Figure 4. Expression of mitochondrial membrane potential, respiration, and dynamic-related proteins in MJD cells. Cells were treated with or without different concentrations of AST for 24 h.


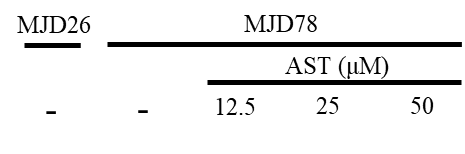


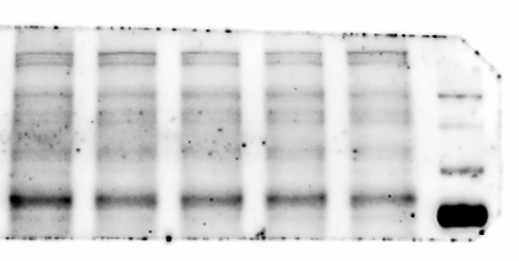

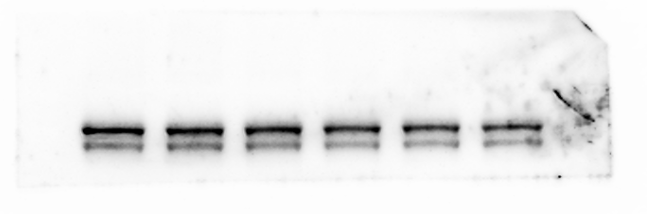


OPA1-L

OPA1-S


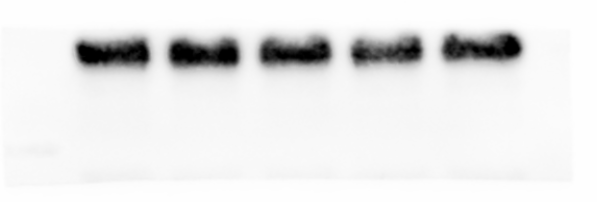

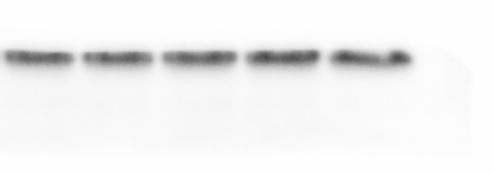

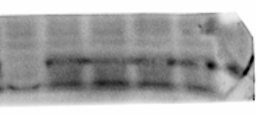

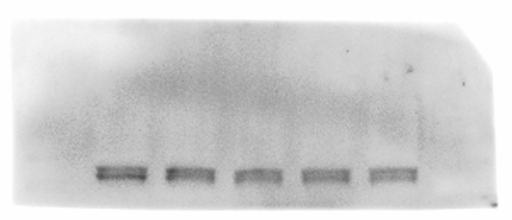

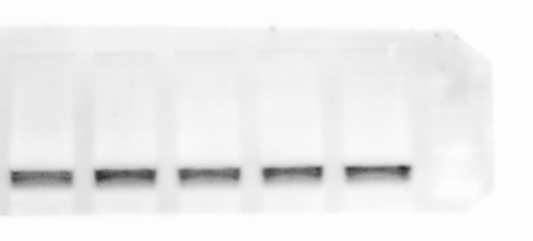


MFN2

GAPDH

Tim23

p-Drp1

Drp1

Fis1
