# Supplementary material for: Astragaloside IV reduces mutant Ataxin-3 levels and supports mitochondrial function in Spinocerebellar Ataxia Type 3
Source: Sci Rep. 2024 Oct 29;14:25979. doi: 10.1038/s41598-024-77763-2 (PMC11522510; doi:10.1038/s41598-024-77763-2)
Supplement: Supplementary file 2 — Supplementary Material 2 [file 41598_2024_77763_MOESM2_ESM.docx]

**Supplement Information (SI)**

Astragaloside IV reduces mutant ataxin-3 levels and supports mitochondrial function in spinocerebellar ataxia type 3

Yongshiou Lin^1^, Wenling Cheng^1^, Juichih Chang^2,3✝^, Yuling Wu^4^, Mingli Hsieh^5^, Chinsan Liu^1,6,7,8*^

Scientific Reports

^✝^The co-first author contributed equally to this work

*Correspondence: Chin-San Liu, Department of Neurology, Changhua Christian Hospital, 7F., No.235, Syuguang Rd., Changhua, Taiwan

Email: liu48111@gmail.com; Tel.: +886-4-7238595#4751

**Methods:**

An aliquot of 20 ng of DNA from cells was subjected to quantitative PCR, which was performed using Roche LightCycler 480 Instruments and Roche Diagnostics LightCycler 480 SYBR Green I Master (Basel, Switzerland). The primers were Forward 5'-AACATACCCATGGCCAACCT-3' and Reverse 5'-AGCGAAGGGTTGTAGTAGCCC-3' for mtDNA-L1 and Forward 5'-GAAGAGCCAAGGACAGGTAC-3' and Reverse 5'-CAACTTCATCCACGTTCACC-3' for β-globin. The relative mtDNA copy number was measured by normalizing the crossing points in the quantitative PCR curves between L1 and β-globin genes.

Autophagic flux in MJD cells

A Premo™ Autophagy Tandem Sensor red fluorescent protein (RFP)-green fluorescent protein (GFP)-LC3B Kit (Cat# P36239, Thermo Fisher Scientific, MA, USA) was utilized to monitor autophagic flux in MJD cells. This kit allowed for enhanced analysis of the maturation process from autophagosome to autolysosome. By using an acid-sensitive GFP alongside an acid-insensitive RFP, the transition from the neutral pH of the autophagosome to the acidic pH of the autolysosome can be observed through the specific loss of GFP fluorescence, leaving only red fluorescence. Chloroquine was used to block autophagic flux, which causes the accumulation of GFP- and RFP-positive vesicles as a positive control. Flow cytometry was used to measure the ratio of red-to-green fluorescence intensity in cells by detecting GFP fluorescence with the FL1 channel and RFP fluorescence with the FL2 channel. (Cytomics FC500 flow cytometry - Beckman Coulter, Inc., Brea, CA, USA; fluorescence excitation/emission maxima: GFP = 488/509 nm; RFP = 555/584 nm) to indicate the change of intracellular autophagolysosomes.


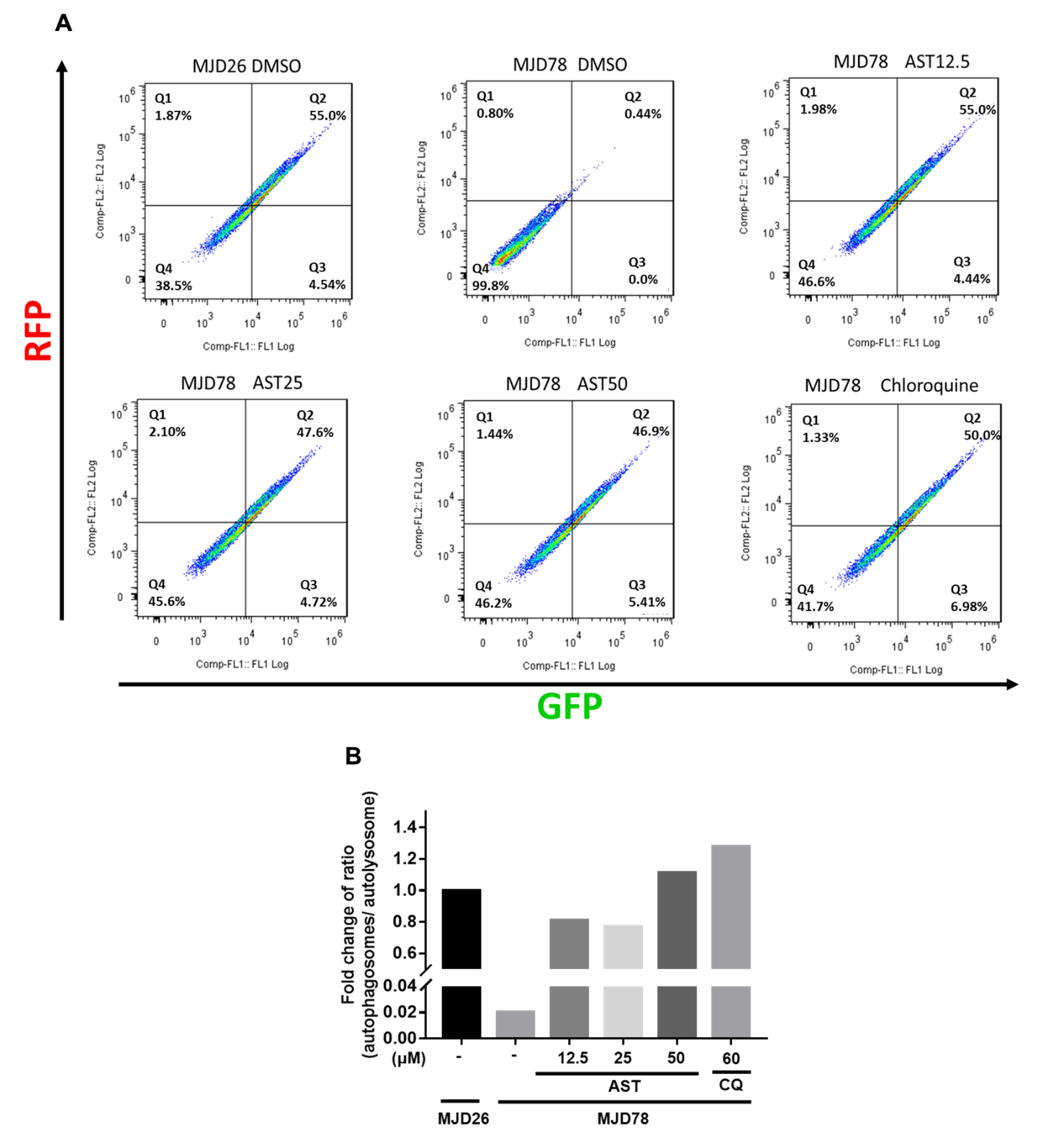


Supplementary Figure 1. Autophagy expression in Machado-Joseph Disease cells after 24-hour treatment with vehicle of DMSO, astragaloside IV with different concentrations or analysis of positive control, chloroquine (CQ). (A) The various stages of autophagy (through LC3B protein localization) was detected by a flow cytometry using the commercially available Premo Autophagy Tandem Sensor RFP-GFP-LC3B Kit. This sensor, which integrated red fluorescent protein (RFP) and green fluorescent protein (GFP), exploited the pH variation between the acidic autolysosome and the neutral autophagosome, as well as the differing pH sensitivities of the two fluorescent proteins, to monitor progression from the autophagosome (GFP fluorescence intensity detected by FL1 channel) to autolysosome (RFP fluorescence intensity detected by FL2 channel). Moreover, CQ was used to block autophagic flux, which caused the accumulation of GFP- and RFP-positive vesicles as a positive control. (B) The transition from autophagosome to autolysosome was quantified by analysis of the ratio of autophagosomes (% of LC3-GFP-RFP positive cells) to autophagosome to autolysosomes (% of LC3-RFP-positive cells).


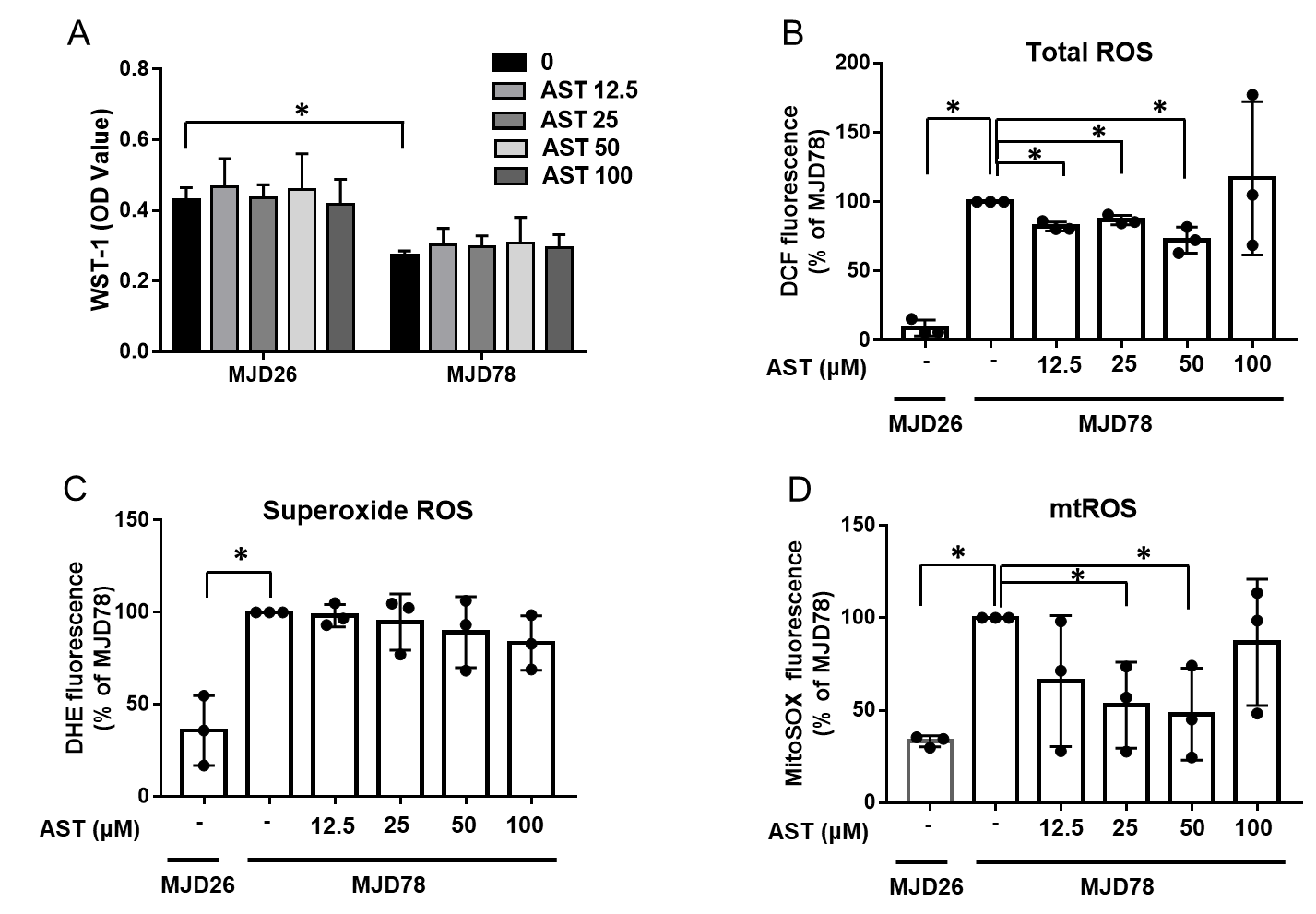


Supplementary Figure 2. Expression of cell viability and oxidative stress in Machado-Joseph Disease cells after treatment with different concentrations of astragaloside IV for 24 hours. (A) Cell viability was assessed using WST-1 (N = 3). Oxidative stress was detected through flow cytometry analysis by performing, (B) DCF staining for intracellular (Total) ROS (N = 3), (C) DHE staining for superoxide ROS (N = 3), and (D) MitoSOX Red staining for mitochondrial superoxide (mtROS) (N = 3). * p < 0.05 was considered significant. Abbreviation: astragaloside IV, AST; Machado-Joseph Disease, MJD; SK-N-SH with 26 CAG repeats, MJD26; SK-N-SH with 78 CAG repeats, MJD78.


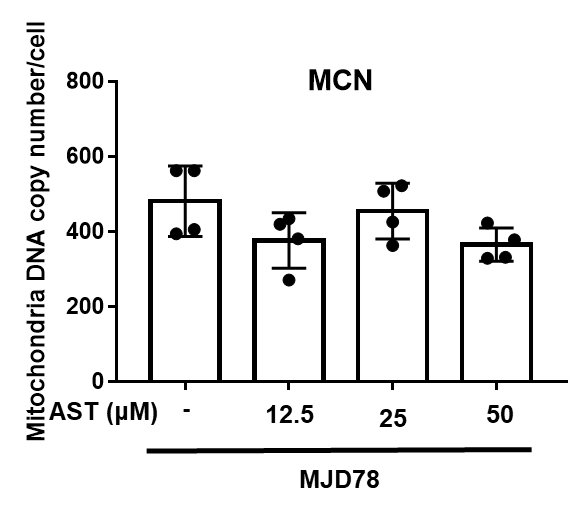


Supplementary Figure 3. Expression of cell mitochondrial DNA (mtDNA) copy number in Machado-Joseph Disease cells after treatment with different concentrations of astragaloside IV for 24 hours. Data are presented as the means ± SDs of four independent experiments.
